# Application of delayed luminescence method on measuring of the processing of Chinese herbal materials

**DOI:** 10.1186/s13020-018-0202-0

**Published:** 2018-08-25

**Authors:** Mengmeng Sun, Wen-Te Chang, Eduard Van Wijk, Min He, Roeland Van Wijk, Mei Wang

**Affiliations:** 1 Leiden University European Center for Chinese Medicine and Natural Compounds, Institute of Biology, Leiden University, Sylviusweg 72, 2333 BE Leiden, The Netherlands; 2 Department of Chinese Pharmaceutical Sciences and Chinese Medicine Resources, College of Chinese Medicine, China Medical University, 91, Hsueh-Shih Road, Taichung, 40402 Taiwan; 3 SU BioMedicine, Postbus 546, 2300 AM Leiden, The Netherlands; 4 Meluna Research, Koppelsedijk 1-a, 4191 LC Geldermalsen, The Netherlands; 5 Changchun University of Chinese Medicine, No. 1035, Boshuo Rd, Jingyue Economic Development District, Changchun, 130117 China; 6 Sino-Dutch Centre for Preventive and Personalized Medicine, Gasthuislingelaan 33, 4002 AG Tiel, The Netherlands

**Keywords:** Delayed luminescence, Chinese herbal medicine, Processing, Quality control

## Abstract

**Background:**

Based on the principle of tradition Chinese medicine, the processing refers to various techniques that alter the overall properties of herbal materials to meet the requirements of therapeutic applications. However, the standards of quality control and scientific standard operation protocol for processing manufacturing are largely unknown and there is a huge demand for the development of scientific tools for evaluating the quality during and after the processing. The key challenge in evidence-based medicine is to characterize the processing of herbal materials from system-based perspective.

**Methods:**

Delayed luminescence (DL) as a rapid, direct, systemic tool was used to characterize the properties of raw and processed materials of Rehmanniae radix and Ginseng radix et rhizome. Hyperbolic function was used to extract four parameters from DL curves of herbal materials. Statistical tools, including one-way analysis of variance and principal component analysis, were used to differentiate raw and processed herbal materials.

**Results:**

Our results showed DL properties were able to reliably identify raw and processed materials of Rehmanniae radix and Ginseng radix et rhizoma, respectively. In addition, the results indicated that after four cycles of processing for Rehmanniae radix, there was no much significant change in DL parameters which resembles the results obtained from chemical analyses (after five cycles) using ^1^HNMR and gas chromatography–mass spectrometry in previous studies.

**Conclusion:**

DL may serve as a fast, robust and sensitive tool for evaluating processing on herbs and may be used as part of a comprehensive platform for assessing the quality of herbal materials.

**Electronic supplementary material:**

The online version of this article (10.1186/s13020-018-0202-0) contains supplementary material, which is available to authorized users.

## Background

In Chinese herbal medicine (CHM), the processing refers to various techniques that alter the properties of herbal materials to meet the requirements of therapeutic applications [1, 2]. It includes basic methods such as removing impurities, washing, cutting, drying, as well as steaming, boiling, calcining and stir frying of herbal materials to increase solubility, remove unpleasant smells, reduce toxicity, decrease side-effects, enhance the pharmacological efficacy and stabilized, and modify the therapeutic property [1, 2]. Based on the principle of Chinese medicine, pharmaceutical actions of CHM are traditionally classified according to the patient’s responses, resulting in descriptive properties of herbal materials such as taste and warm/cold [3]. Processing has a close relationship with the therapeutic properties. For example, according to Chinese medicine principle, raw Rehmanniae radix is considered to have “sweet and bitter” with “cold” properties and can be used to treat “heat” syndromes; but the processed Rehmanniae radix has been provided with “sweet” and “warm” properties and can be used to treat “cold” syndromes [4]. In addition, white Ginseng radix et rhizoma is considered to have the property “warm”, but this property may be enhanced in its processed product named red Ginseng radix et rhizome [5]. The different therapeutic properties of these herbs are also recognized in the Chinese Pharmacopoeia 2015 to the extent that there are now separate monographs for raw and processed Rehmannie radix, as well as white and red Ginseng radix et rhizoma, reflecting their differences in therapeutic applications [5, 6]. Moreover, modern research has indicated that chemical components can be changed by processing and then may induce different therapeutic properties in these herbal materials [6, 7].

In current CHM practice, almost all the herbal materials are strictly required to be properly processed before using for therapeutic applications. Yet, the standards of quality control and scientific standard operation protocol for processing manufacturing are largely unknown and there is a huge demand for the development of scientific tools for fast, robust and sensitive measurements for evaluating the quality during and after the processing. There is a common concept to the processing of herbal materials in CHM: “obeying ancient processing methods” [1, 2]. For instance, the ancient method of “steam with rice wine nine times and dry nine times” was emphasized in the processing of Rehmanniae radix [4]. However, the ancient methods may not suit to modern industrial production and the processing methods for many herbal materials have been already changed [1, 2]. Since lack of objective criteria, non-unified standards are still existed in practice in different locations of China, and the technology of CHM processing mainly depends on the practitioner’s experiences. Therefore, to develop the tools of quality control for herbal materials processing are urgently needed. As a conventional method, chemical analysis is the most common method and has provided many important information to help the quality assessment on CHM processing. For instance, Chang and colleagues have reported that the modification of therapeutic properties of processed Rehmanniae radix was related to the changes of metabolic profiling during the processing [4]. In addition, Raman, mid-infrared and near-infrared spectroscopy as non-destructive and fast measurement tools have been used to discriminated raw and processed plant materials [8–10]. Given the overall changes in CHM processing, comprehensive, systemic and rapid method for quality assessment of CHM processing are still worth to further investigate.

Recently, various luminescence related technologies are widely used in the mechanism of disease research and drug quality control [11–17]. Delayed luminescence (DL) is the long-term decay of weak photon emissions from materials following exposure to light with a wavelength of 400–800 nm [3]. DL provides a comprehensive method for measuring biological systems, including food quality and seed germination [18, 19]. Recently, we used DL to study the features of Chinese herbal materials. The results suggest that specific DL properties can be used to indicate differences in herbal materials prepared under different conditions, including the age of the herb, the grown environments [20, 21]. These differences in DL properties reflect variations in both the bioactive components contained in the herb and the therapeutic properties of herbal materials. Importantly, in our preliminary results, DL was used to distinguish the Acunitum roots (*Aconitum carmichaelii* Debx.) materials processed by different methods [21]. In this study, we measured DL between raw and processed Rehmanniae radix, as well as white and red Ginseng radix et rhizoma in order to further investigate the application of DL in the quality control of CHM processing. The results obtained from our DL measurements show DL properties can indicate the changes in processing of these herbal materials; therefore, DL can provide new insights into the processing of herbal medicines.

## Methods

### Information of experimental design and resources

The information regarding the experimental design, statistics, and resources used in this study are attached in the minimum standards of reporting checklist (Additional file 1).

### Herbal materials

Raw roots of Rehmannia (*Rehmannia glutinosa* Libosch.) were provided by China Medical University (Taichung, Taiwan). For preparing processed Rehmanniae radix, the raw roots were dried in an oven at 55 °C, then the dried roots were soaked in yellow rice wine (Michiu, Taiwan Tobacco & Liquor Corp., Taipei, Taiwan) for 15 min, and steamed with water for 60 min. After that, the processed Rehmanniae radix was dried overnight at 55 °C [4]. This procedure was repeated for nine times to obtain processed Rehmanniae radix from one to nine cycles, respectively [4]. Roots and rhizomes of white and red Ginseng (*Panax Ginseng* C. A. Mey.) were purchased in five batches from TongRenTang Co., Ltd. (Beijing, China). The identities of all herbal samples were verified by Dr. Wen-Te Chang (China Medical University) and were deposited at China Medical University (Taichung, Taiwan).

### Delayed luminescence

#### Sample preparation

Each herbal material was crushed using a model QE-100 grinder (Yili Company, Zhejiang Province, China) and passed through a standard sieve to obtain 150-μm particle. All the herbal materials were kept in a dark, light–tight box containing 35-mm silica gel (Boom BV, Meppel, the Netherlands) at room temperature for 16 h before DL measurements were conducted [21].

#### DL measurement

The procedure to perform DL measurements was described previously [21]. The equipment used to measure DL was provided from Meluna Research (Geldermalsen, the Netherlands) and included a photomultiplier tube (PMT) (type 9558QB; Electron Tubes Enterprises Ltd., Ruislip, UK) vertically positioned on a dark sample chamber kept at 22 °C. The PMT contains a cathode end (51 mm diameter) with sensitivity at 300–800 nm. The PMT was cooled to − 25 °C in order to reduce the dark count rate to 10 counts per second. The DL signal was amplified using a type 9301 fast preamplifier (ORTEC, Oak Ridge, TN). Data were acquired using a personal computer containing a model 6602 counting card (National Instruments, Austin, TX).

Each Rehmanniae radix sample was used to prepare five 1-g samples. Each 1-g sample was placed in a Petri dish (35-mm diameter) and illuminated for 10 s using a model 284-2812 white halogen excitation source (Philips, Germany). For each 1-g sample, the DL signal was measured three consecutive times, resulting in a total of fifteen measurements in the five samples to examine the DL properties of that particular Rehmanniae radix. Each batch of Ginseng radix et rhizoma (1 g) was placed in a Petri dish and illuminated for 10 s using the same white halogen light source, then measured three consecutive times, and a total of fifteen measurements in five batches were used to examine the DL properties of that particular Ginseng materials. The DL decay signature was obtained by recording the number of photon counts in consecutive 0.05-s periods for a total of 60 s, yielding a total of 1200 data points.

#### Data processing and statistical analysis

The DL decay curve for each sample, measured over a 60-s period, was fitted to the following hyperbolic function [22]:

$$I_{\left( t \right)} = \frac{{I_{0} }}{{\left( {1 + \frac{t}{Tau}} \right)^{Beta} }}$$

$$T = \left( {e^{{\frac{1}{ Beta}}} - 1} \right) \times Tau$$

where I_0_ is the initial intensity of the DL curve, Beta is an index factor associated with the rate of DL decay, and Tau and T represents the DL characteristics and decay time, respectively [22]. The R package nnet (version 3.2.2) was used to perform the DL curve-fitting [23]. The average of each DL property from the fifteen measurements was calculated and used to represent the DL signature of each herbal material. One-way analysis of variance (ANOVA) with least significant difference (LSD) post hoc analysis was used to compare the DL properties between raw and processed Rehmanniae radix; differences were considered significant at *p *< 0.05. Principal component analysis (PCA) and Orthogonal projections to latent structures discriminant analysis (OPLS-DA) was used to indicate the level of discrimination between DL properties in Rehmanniae radix samples, using the tools provided in the MetaboAnalyst software package (http://www.metaboanalyst.ca) [24]. A two-tailed, unpaired Student’s *t* test (SPSS version 23.0) was used to compare the DL properties between white and red Ginseng radix et rhizoma; differences were considered significant at *p *< 0.05.

## Results

**Fig. 1:**
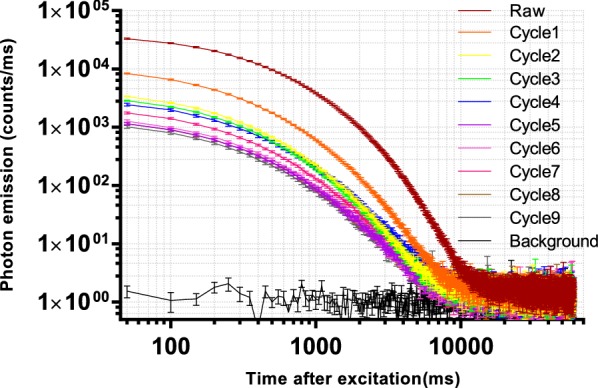
DL decay curves for pooled samples from Rehmanniae radix samples. Data are plotted as the mean ± SEM. Note that the data are plotted on a log–log scale

**Fig. 2:**
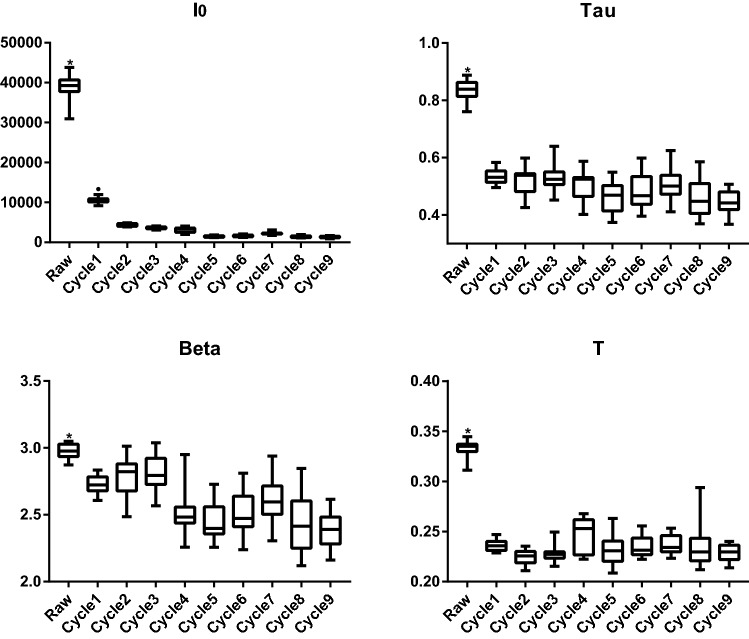
Box plot summarizing the four DL properties measured in the Rehmanniae radix samples. I0 is the initial intensity of the DL curve, Beta is an index factor associated with the rate of DL decay, and Tau and T represents the DL characteristics and decay time, respectively. *p < 0.05 between raw material and all materials in processing cycles; ^*●*^*p* < 0.05 between material in processing cycle 1 and materials in the other processing cycles (one-way ANOVA with LSD)

**Fig. 3:**
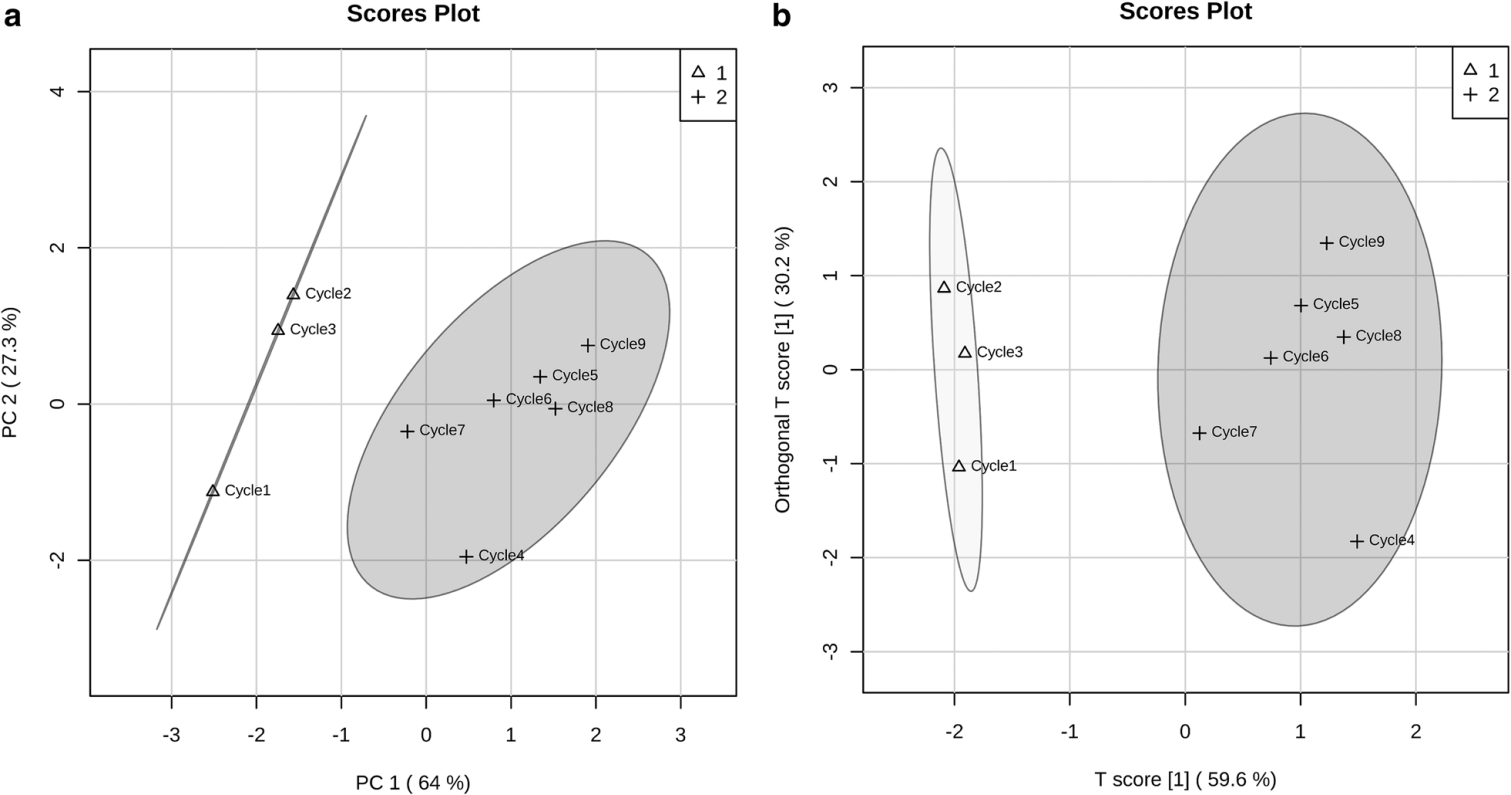
PCA and OPLS-DA scores obtained from the DL data. **a** PCA and **b** OPLS-DA score plots of the DL data obtained from processed Rehmanniae radix samples, showing two separated clusters. The separated clusters were marked with a “△” (cluster 1) or a “+” (cluster 2) symbol, in which cycles 1–3 belongs to one cluster and cycles 4–9 belongs to another cluster

According to the ancient description for the processing of Rehmanniae radix, “steaming and drying for nine cycles”, has been regarded as the standard method [4]. More scientific studies need to be established to understand the working principle of this type of processing. Previous studies suggested that differences in chemical profiles of processed Rehmanniae radix became smaller after five processing cycles (Additional file 2: Fig. S1) [4, 25, 26]. In this study, DL has been used to measure raw and processed Rehmanniae radix samples in order to investigate the processing approach. We pooled the data from fifteen measurements to plot DL decay curves in specific Rehmanniae radix samples. Figure 1 illustrates the different DL kinetics of Rehmanniae radix samples using a log–log scale (A linear scale in Additional file 3: Fig. S2). Raw Rehmanniae radix samples show significantly different DL curves compared with processed materials, and differences generally become smaller after multiple processing. The DL spectra of Rehmanniae radix samples are illustrated using raw material as an example in Additional file 4: Fig. S3. Next, four parameters were derived from these DL curves, these four parameters were compared between all Rehmanniae radix samples using a one-way ANOVA. The results are summarized in Fig. 2. Figure 2 reveals that all four parameters (T, I_0_, Beta and Tau) can be used to differentiate between raw and processed Rehmanniae radix samples. Only I_0_ can be used to distinguish between cycle 1 and the other cycles in processed Rehmanniae radix samples. To further display the differences between processed Rehmanniae radix samples in DL properties, an unsupervised PCA approach was used to obtain a focused view of the variance in the four DL parameters. Figure 3a shows the PCA score plot using the four DL parameters in which PC1 and PC2 account for 64% and 27.3% of the total variance, respectively. This plot shows that the DL properties of the first three processed cycles and the last six processed cycles generally form distinct clusters, with the seventh processed cycle misclassified as belonging to the group on the left side of Fig. 3a. Therefore, a supervised clustering approach (OPLS-DA; Fig. 3b) was further performed to make the optimal separation. OPLS-DA evaluation with cross-validation revealed predictive accuracy of 0.88 and goodness-of-fit (R^2^) of 0.93. The results obtained from the OPLS-DA analysis revealed a better classification effect compared to the results obtained from the PCA analysis. The data show that the differences in DL properties of processed Rehmanniae radix became smaller after the fourth processing cycle. Thus, DL shows similarities in identified effects to indicate the processing of Rehmanniae radix compared to chemical analyses (Additional file 2: Fig. S1) [4, 25, 26].

**Fig. 4:**
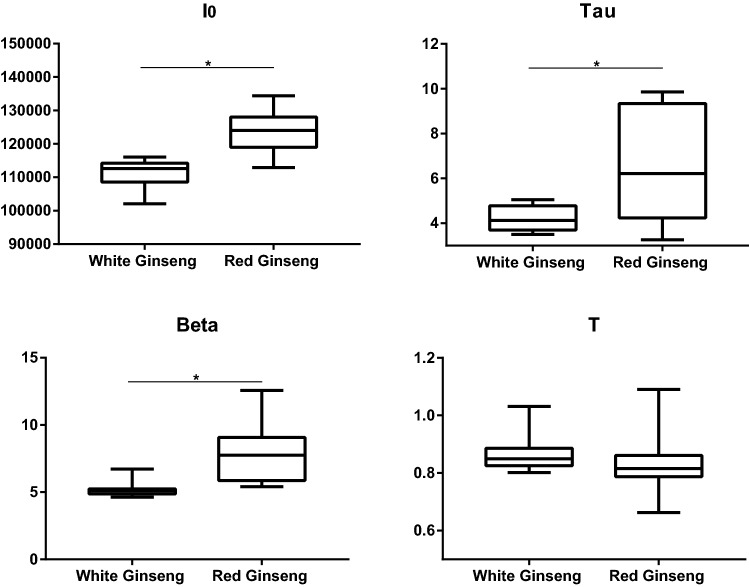
Box plot summarizing the four DL properties measured in the Ginseng radix et rhizoma samples. *p < 0.05 (two-tailed, unpaired Student’s t-test)

As a next step for further examination of the identification effects of DL in herbal materials processing, commercial white and red Ginseng radix et rhizoma were studied. The DL spectra of Ginseng samples are summarized in Additional file 4: Fig. S3. The significant differences in most DL parameters were found between white and red Ginseng radix et rhizome (Fig. 4), which indicates DL may be suitable in measurements for more processed herbal materials.

## Discussion

Our data indicates that DL may reflect the changes caused by processing in Rehmanniae radix and Ginseng radix et rhizoma. The molecular absorption of excitation energy determines the dynamics of the subsequent DL emission [27]. DL properties can be influenced by changes of molecular conformations and interactions such as forming of hydrogen bonds and carbon-to-nitrogen ratio [20, 21], resulting in the radiant transfer of energy from one excited molecule to another, causing a change in the herb’s DL kinetics [3]. Processing can lead to significant chemical changes [28]. For example, Rehmanniae radix is rich in polysaccharides, which can be hydrolyzed to monosaccharides (e.g. galactose and glucose) during the processing [4]. In addition, the contents of catalpol, raffinose, and stachyose gradually decrease by processing in Rehmanniae radix [4]. Moreover, changes in concentration of acidic polysaccharides and structural conversions of ginsenosides are detected in white and red Ginseng radix et rhizome [6]. Since these primary and secondary metabolites in Rehmanniae radix and Ginseng radix et rhizoma can interact with molecular conformations and interactions in an overall level [29], the DL properties show significant changes during processing. Interestingly, DL properties of processed Rehmanniae radix can be classified in PCA and OPLS-DA analyses. It indicates that the DL characteristics became stable after the fourth processing cycle. This result is similar with previous chemical analyses which indicated that the chemical components were stable after the fifth processing cycle using plant metabolomics tools such as ^1^HNMR and gas chromatography–mass spectrometry (GC–MS) [4, 25, 26]. Plant metabolomics provides a relatively comprehensive analysis of various compounds in biological systems based on specific platforms [30], and it has been considered as a suitable tool for herb quality control in a system level [31]. Similar to plant metabolomics, DL is a highly sensitive systemic measurement that reflects the overall properties of a biological system [32, 33]. Therefore, DL can provide a close approximation of the system level structures identified by using plant metabolomics. However, metabolomics analyses are not able to evaluate the entire integral profile of chemical components since diversity of various molecular and the limitation in measurement technology platforms. In contrast, DL can measure directly the herbal materials and reflect systemic properties of herbs. Therefore, combing DL properties and herbal metabolites may provide a comprehensive work platform which may reveal new insight into herbal quality control in the future.

In the principle of Chinese medicine, the therapeutic actions of herbs are interpreted by a conceptual description of herbal properties such as taste and warm/cold etc. [3]. This type of description is based on practice experience which aiming the treatment of the human body as a whole [34, 35]. Therefore, based on the Chinese medicine’s principle the therapeutic actions are modified after the processing procedure [5, 6]. Modern chemical and biological research may reveal the changes of metabolites after the processing and therefore the pharmacological effects [6]. However, the therapeutic effects of herbs are not simply related to the identified components, as other chemical compounds within herbs can combine synergistically with bioactive components to form overall therapeutic effects [36]. Therefore, comprehensive strategies should be considered in quality control of herbal materials processing to further explain the effects of processing on the pharmacological actions of herbs. In this aspect, to explore novel technology on quality assessment based on therapeutic effects is very important. DL measurement is a new tool, need further to explore for evaluating the changes of therapeutic properties caused by herbal processing. The results of this study provides a suitable foundation for follow-up studies. Further studies can include more herbs, and additional platforms for investigation the bioactivity of raw and processed herbal materials. In brief, DL is a new technique for studying CHM, thereby facilitating the move toward the comprehensive quality control in herbal materials processing.

## Conclusion

The data presented in this article shows that DL properties from raw and processed herbal materials can be distinguished significantly. DL measurements show similar results to differentiate raw and processed Rehmanniae radix compared to chemical analysis using plant metabolomics [4, 25, 26]. The results indicate that after four cycles of processing for Rehmanniae radix, there is no much significant change in DL parameters which resembles the results obtained from chemical analysis (after five cycles). As multi-processing method has been recognized to treat raw Rehmanniae radix in both Chinese and Vietnamese pharmacopoeias [1], further research should be performed to validate whether DL can identify raw and processed Rehmanniae radix prepared under different processing methods. Since we are able to detect different DL characters in different processed Aconitum roots prepared using different methods [21], it is highly possible that different processing method will lead to different DL characteristic parameters, however, further confirmation for this predication required additional research. Finally, DL provides a rapid, direct, systemic tool for analyzing overall properties of herbal processing. It is a promising new technology to measure quality control in herbal materials [37].

## Additional files

**Additional file 1.** Minimum standards of reporting checklist.

**Additional file 2: Fig. S1.** Dendrogram of the hierarchical cluster analysis of chemical profiles of raw and processed Rehmanniae radix samples. A–C indicates three independent batches of raw and processed Rehmanniae radix samples in previous study [4]. ^1^HNMR and Fourier transform (FT)-mass spectrometry were used to obtain chemical profiles of these samples [4]. Chemical data of these samples was used to perform hierarchical cluster analysis (Distance Measure: Spearman; Clustering Algorithm: Ward).

**Additional file 3: Fig. S2.** DL decay curves for pooled samples from Rehmanniae radix samples. Data are plotted as the mean ± SEM. Note that the data are plotted on a linear scale.

**Additional file 4: Fig. S3.** Spectral distribution of DL emission of Rehmanniae radix and Ginseng radix et rhizome.

## Abbreviations

DL: delayed luminescence
CHM: Chinese herbal medicine
ANOVA: one-way analysis of variance
LSD: least significant difference
PCA: principal component analysis
PLS-DA: orthogonal projections to latent structures discriminant analysis
GC–MS: gas chromatography–mass spectrometry
