# Supplementary material for: Application of delayed luminescence method on measuring of the processing of Chinese herbal materials
Source: Chin Med. 2018 Aug 25;13:43. doi: 10.1186/s13020-018-0202-0 (PMC6109338; doi:10.1186/s13020-018-0202-0)
Supplement: Supplementary file 2 — Additional file 2: Fig. S1. Dendrogram of the hierarchical cluster analysis of chemical profiles of raw and processed Rehmanniae radix samples. A–C indicates three independent batches of raw and processed Rehmanniae radix samples in previous study [4]. 1HNMR and Fourier transform (FT)-mass spectrometry were used to obtain chemical profiles of these samples [4]. Chemical data of these samples was used to perform hierarchical cluster analysis (Distance Measure: Spearman; Clustering Algorithm: Ward). [file 13020_2018_202_MOESM2_ESM.pdf]

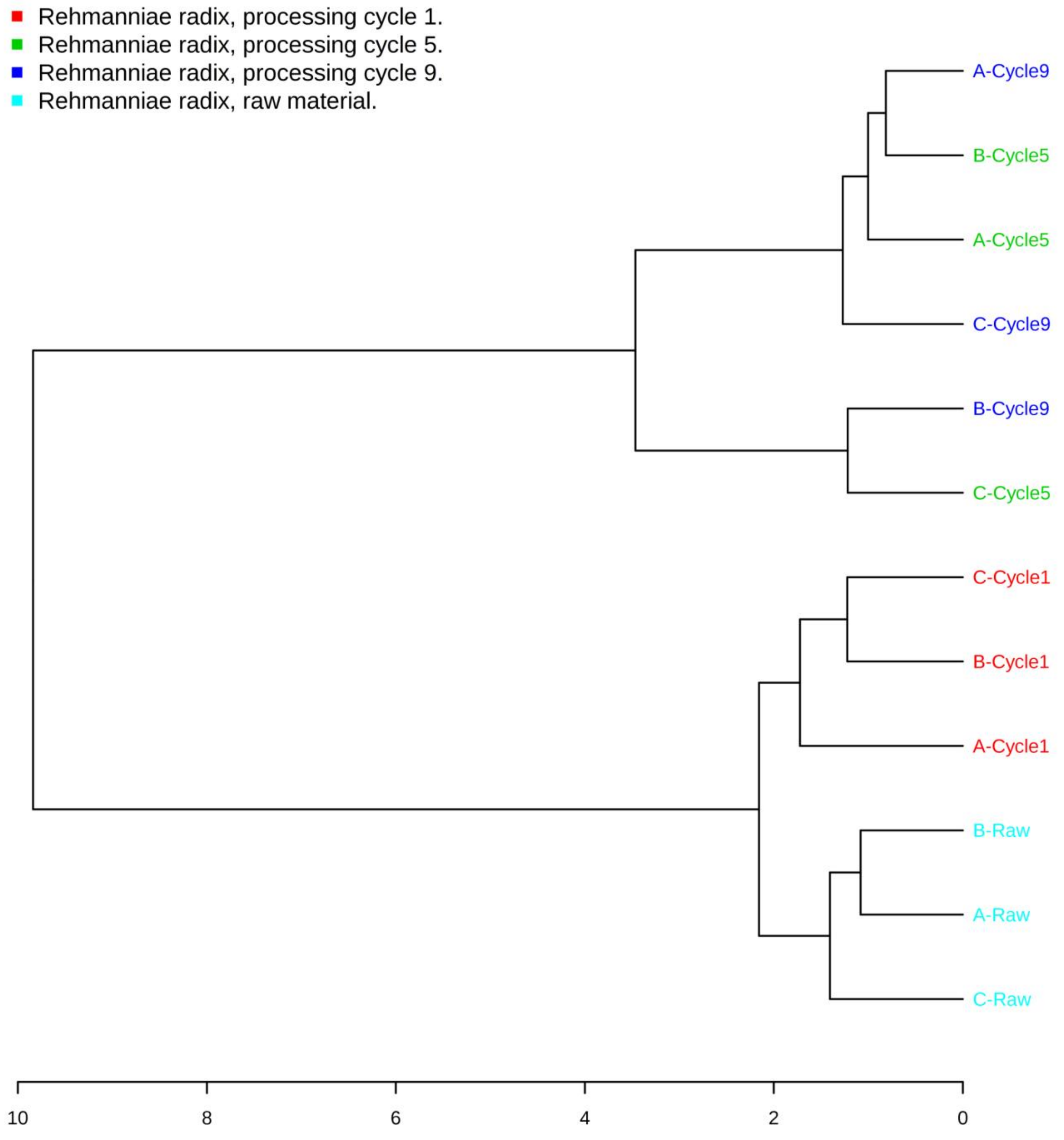

Rehmanniae radix, processing cycle 1.
Rehmanniae radix, processing cycle 5.
Rehmanniae radix, processing cycle 9.
Rehmanniae radix, raw material.
A-Cycle9
B-Cycle5
A-Cycle5
C-Cycle9
B-Cycle9
C-Cycle5
C-Cycle1
B-Cycle1
A-Cycle1
B-Raw
A-Raw
C-Raw
10
8
6
4
2
0
