# Supplementary figures and images for: Application of delayed luminescence method on measuring of the processing of Chinese herbal materials
Source: Chin Med. 2018 Aug 25;13:43. doi: 10.1186/s13020-018-0202-0 (PMC6109338; doi:10.1186/s13020-018-0202-0)

Raw

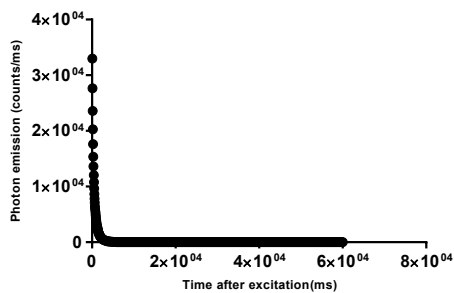

Cycle1

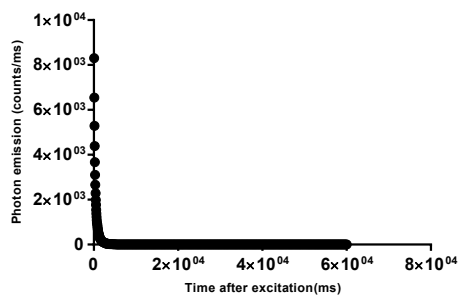

Cycle2

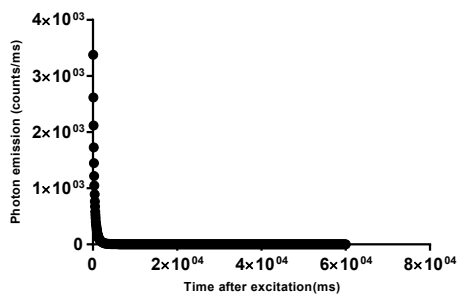

Cycle3

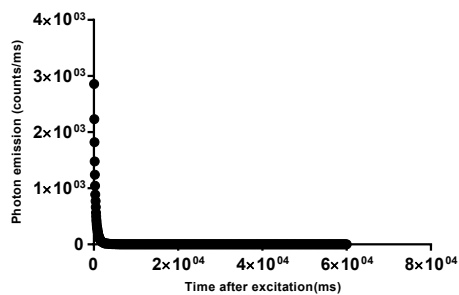

Cycle4

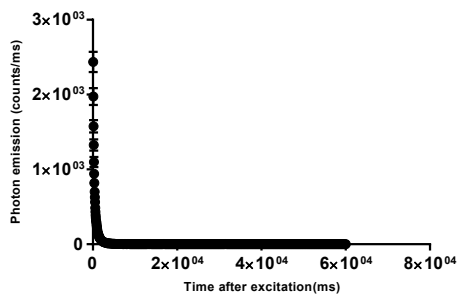

Cycle5

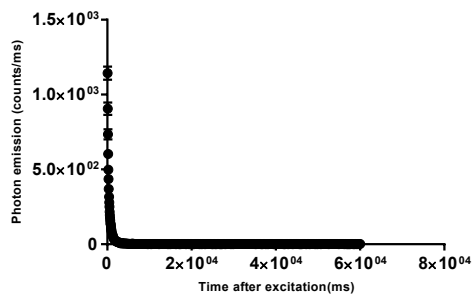

Cycle6

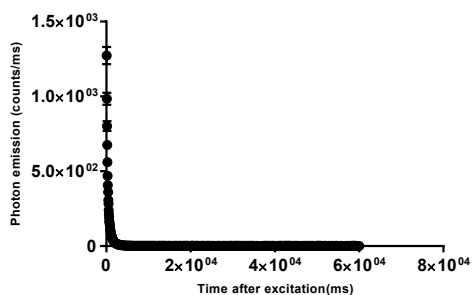

Cycle7

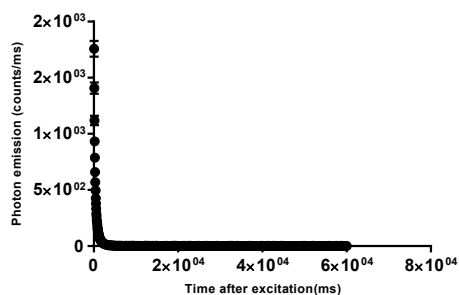

Cycle8

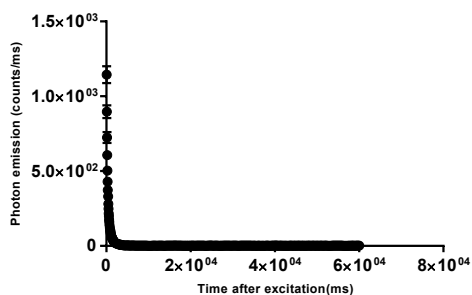

Cycle9

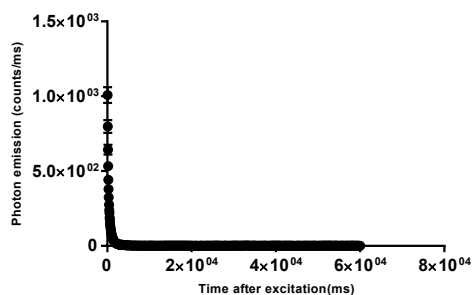

Supplement: Supplementary file 3 — Additional file 3: Fig. S2. DL decay curves for pooled samples from Rehmanniae radix samples. Data are plotted as the mean ± SEM. Note that the data are plotted on a linear scale. [file 13020_2018_202_MOESM3_ESM.pdf]

**A****Rehmanniae radix (Raw)**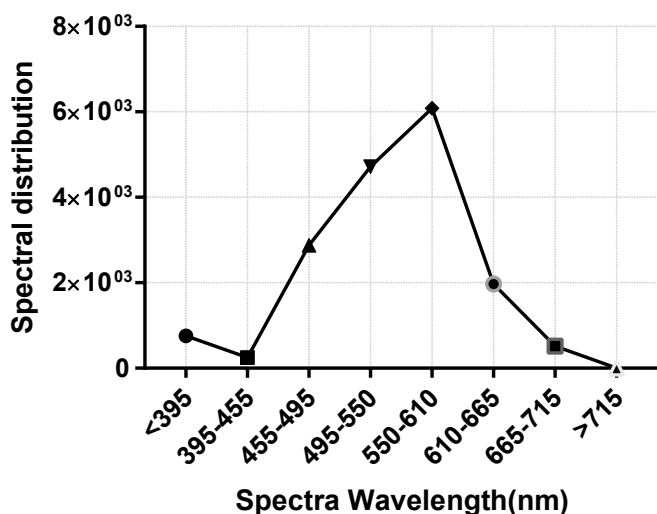**B****White Ginseng**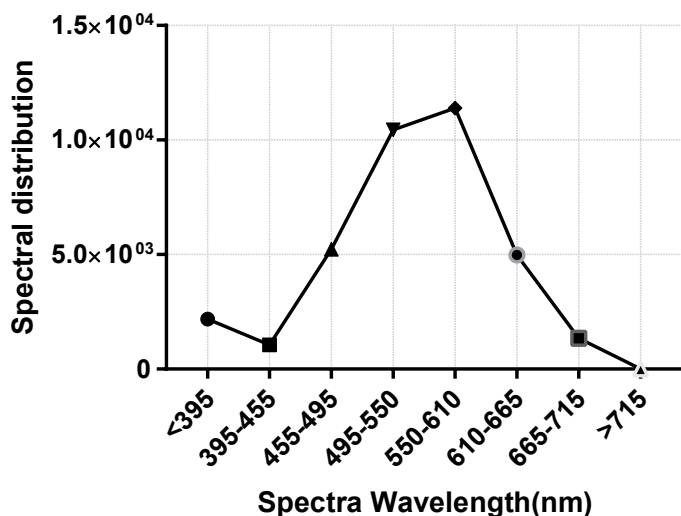**C****Red Ginseng**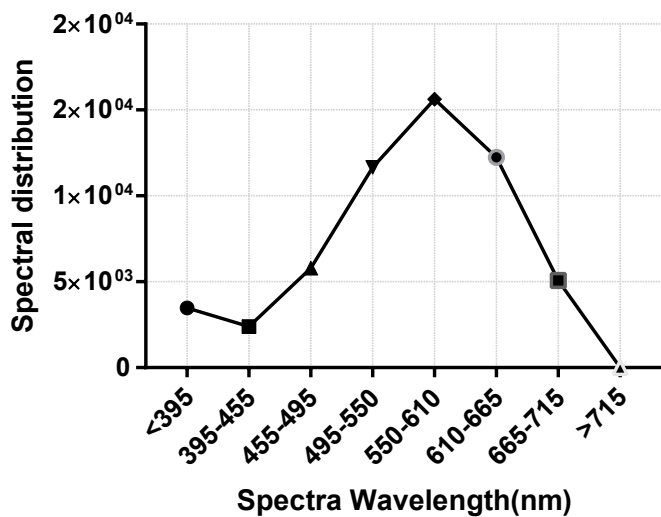

Supplement: Supplementary file 4 — Additional file 4: Fig. S3. Spectral distribution of DL emission of Rehmanniae radix and Ginseng radix et rhizome. [file 13020_2018_202_MOESM4_ESM.pdf]
